# Electromyography of the External Anal Sphincter during Micturition and Electrophysiological Bulbocavernosus Reflex in Healthy Spayed Female Canines

**DOI:** 10.1155/2023/3822212

**Published:** 2023-01-30

**Authors:** Nicha Mongkolrat, Areerath Akatvipat, Phitchaya Saenubol, Pachara Pornnimitara, Sukolrat Boonyayatra, Siam Tongprasert

**Affiliations:** ^1^Department of Companion Animals and Wildlife Clinic, Faculty of Veterinary Medicine, Chiang Mai University, Chiang Mai 50100, Thailand; ^2^Department of Food Animal Clinics, Faculty of Veterinary Medicine, Chiang Mai University, Chiang Mai 50100, Thailand; ^3^Department of Veterinary Clinical Sciences, College of Veterinary Medicine, Long Island University, Brookville, NY, USA; ^4^Department of Rehabilitation Medicine, Faculty of Medicine, Chiang Mai University, Chiang Mai 50100, Thailand

## Abstract

This study aimed to find methods and interferences and illustrate the pattern of external anal sphincter (EAS) electromyography (EMG) during micturition and to determine reference intervals of electrophysiological bulbocavernosus reflex (EBCR) by using robust statistical methods in healthy spayed female canines. Ten healthy spayed female canines (no breed restriction) with a body weight of 11.3–18 kg were enrolled. EAS EMG during micturition and the EBCR test were performed under light general anesthesia. Altogether 25 out of 34 EAS EMG showed a similar pattern, including low-amplitude high-frequency bursting pattern before voiding, medium- or high-amplitude low-frequency bursting pattern at the beginning of voiding, oscillate medium- and/or high-amplitude low-frequency bursting with a low-amplitude high-frequency bursting pattern during voiding, and high-amplitude high-frequency bursting pattern at the end of voiding. An average of 100 consecutive stimulations of EBCR for one cycle were performed in each dog and another cycle was repeated to ensure reproducibility. The lower and upper limits of the reference interval of EBCR onset latency values and EBCR mean amplitude values were calculated using both standard and robust methods with untransformed and transformed Box-Cox data. The EBCR onset latency was between 13.85 and 27.44 milliseconds, whereas the EBCR mean baseline to peak amplitude was not transformed with Box-Cox transformation. All EBCR compound muscle action potentials started with a negative sharp wave, which tapers from the baseline in the upward direction, showing an upturned bell-shaped curve. In conclusion, this study was possibly the first to examine the method and provide the electrographic pattern of EAS EMG during micturition and reference intervals of EBCR onset latency in spayed female dogs, which may serve as baseline information to help veterinarians differentiate healthy from diseased dogs. Further studies should compare normal dogs and dogs with lower urinary tract abnormalities at different lesion locations.

## 1. Introduction

Adult neurogenic lower urinary tract dysfunction refers to the incoordination of the bladder, neck of the bladder, and urinary bladder sphincter activities that occur due to damage at any site of the micturition pathway. The pathway consists of the supraspinal pathway, segmental spinal cord reflex, and pelvic, hypogastric, and pudendal nerves [1].

In canines, secondary spinal cord injury is the most common cause of neurogenic lower urinary tract dysfunction, which leads to poor quality of life, difficulty in long-term care, and death [2, 3]. Neurogenic lower urinary tract dysfunction remains one of the challenging problems due to the insufficiency of effective treatment and objective measurements of neurological recovery in veterinary medicine [4, 5]. Detrusor sphincter dyssynergia (DSD) is one of the neurogenic lower urinary tract dysfunctions that may lead to kidney damage due to dangerously high spikes in bladder pressure as detrusor contraction concurrent with an involuntary contraction of the urethral and/or periurethral striated muscle, which could prevent the flow of urine. Measurements of both detrusor and external urethral sphincter (EUS) functions are required to diagnose DSD [6]. In human medicine, electromyography (EMG) of the sphincter during micturition is one of the diagnostic tools for DSD, and the neurological cause of a patient's voiding dysfunction can be essentially ruled out when a normal finding is obtained by EMG [1]. However, EUS EMG is very invasive, uncomfortable, and hard to perform in animals. To facilitate the ease of application, the external anal sphincter (EAS) becomes the target muscle for detecting electrical activity during micturition in veterinary research because both EAS and EUS are innervated by the pudendal nerve branches and urethral stimulation by the passage of urine through the urethra results in the increased electrical activity of EAS as it contracts to prevent stool leakage, which is referred to as the urethro-anal reflex [7, 8]. A previous study indicated that EUS EMG patterns between mice and rats were different [9]. Moreover, one study revealed the different bundles of circular sphincteric muscle at the urinary bladder between a female dog and a woman [10], which suggests that the functional study of EUS EMG or EAS EMG in humans may not apply to dogs. Therefore, further studies on each animal species are required. Our research team in this study is interested in examining the methods and precautions and finding the pattern of EAS EMG during micturition in canine species.

The electrophysiological bulbocavernosus reflex (EBCR) is also one of the neurophysiological tests in human patients with suspected adult neurogenic lower urinary tract dysfunction [11]. Contraction of the bulbocavernosus muscle is elicited by stimulating the urogenital region. A previous study on the EBCR in a canine model suggested that the EBCR test has the potential to become a diagnostic tool for the sacral reflex in small animals [12, 13]. The mean latency and standard deviation of EBCR in male and female Beagles were 24.42 ± 1.09 (range from 17.6 to 28.8) and 22.26 ± 1.92 (range from 18.99 to 25.69) milliseconds (ms), respectively [12, 13]. Some studies in the past have reported the effects of sex hormones on neuromuscular function [14–17]; thus, this is possibly the first study of EBCR in spayed female canines, owing to which estrous cycle was not affected.

Theoretically, the classic method revealed that at least 120 healthy individuals are needed to find the reference interval and apply nonparametric estimates of the 95% reference interval, which consumes extensive labor, cost, and time. Fortunately, robust statistical methods have been introduced to resolve the limitations of classic methods. These methods can help researchers to obtain a good estimate of reference intervals with a small number of samples. It is currently widely used in various disciplines, including medical and neuroscience [18–20]. Therefore, only 10 healthy spayed female canines were used in this study.

The objectives of this study were (1) to find the method and interference and illustrate the pattern of EAS EMG during micturition in healthy spayed female canine under light general anesthesia and (2) to determine the reference intervals of the EBCR in healthy spayed female canines by augmenting the power of the dataset with robust statistical methods.

## 2. Materials and Methods

### 2.1. Subject Selection

Healthy spayed female dog volunteers with no breed restriction were enrolled in the study. The inclusion criteria were healthy spayed female dogs aged between 8 months and 7 years with no history of distemper virus infection or any diseases that might affect the nervous system and without any neuromuscular diseases. Additionally, dogs with clinical signs of infectious disease, systemic disease, and neurological abnormality, and those with abnormal findings in the urine were excluded from the study.

All subjects underwent physical examination, complete blood count, blood chemistry, neurological examination, and urinalysis to meet inclusion criteria.

### 2.2. Ethics Statement

All animals in this study were healthy spayed female dog volunteers and had owner-authorized consent forms. All animal procedures were approved by the Faculty of Veterinary Medicine, Chiang Mai University Animal Care and Use Center (FAC-ACUC No. S30/2563).

### 2.3. Study Design

The owner was informed to withhold the food before the study for at least 6 hours. Dogs were administered anesthesia with dexmedetomidine 1 mcg/kg intravascularly and dexmedetomidine 1 mcg/kg intramuscularly. Continuous propofol at a sufficient dosage of 2–6 mg/kg was administered intravascularly to maintain anesthesia depth in stage 3 plane 1, which was characterized by central eye position, presence of palpebral reflex, and jaw tone without movement. Oxygen supplementation and monitoring of oxygen saturation, heart rate, and blood pressure were continuously performed. When a stable anesthesia depth was achieved, the dog was placed on the right lateral recumbency. The hair around the vulva and anus was clipped, and the skin was scrubbed with diluted 2% chlorhexidine and then cleansed with a sterile irrigation solution. EAS EMG during micturition was recorded before performing the EBCR. After the experiment, Novacilan® (50% sodium phenyl dimethyl pyrazolone methyl aminomethane sulphonate monohydrate (Dipyrone); Union Drug Laboratories Ltd., Thailand) 0.5 ml was administered intravascularly to all subjects for anti-inflammation, and Antisedan® (Atipamezole hydrochloride; Zoetis, United States) 20 mcg/kg was administered intravascularly to reverse the effect of dexmedetomidine.

### 2.4. Electromyography of the External Anal Sphincter during Micturition

**Figure 1:**
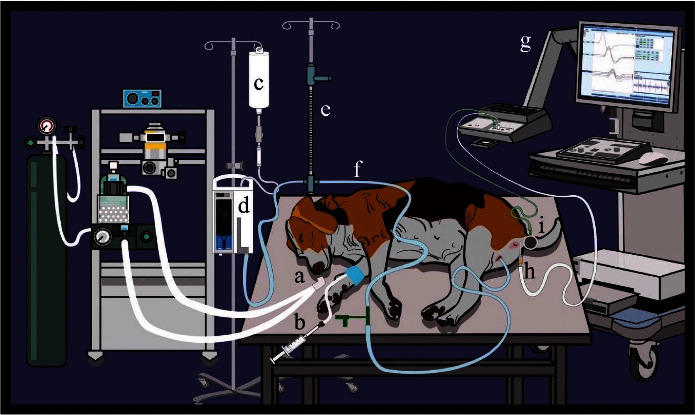
Set up instruments, EMG workstation, and animal position during EAS EMG examination. Oxygen was given to the dog (a), and an intravenous catheter was placed on the cephalic vein and connected with an extension line and propofol syringe (b). The lactated Ringer's solution bottle (c) was connected to the infusion pump (d), central venous manometer (e), and 5-Fr polyvinyl chloride feeding tube catheter (f), which retrogradely infused fluid to the urinary bladder at a rate of 20 mL/min. The concentric needle electrode was placed at 30° left lateral side with a 1 cm distance parallel to the anal orifice (h). A ground electrode was placed at the tail base (i). EAS EMG was performed using Dantec® Keypoint® G4 EMG/NCS/EP workstation (g).

A 5-Fr polyvinyl chloride feeding tube catheter was inserted into the bladder through the urethra, and urine was evacuated. Micturition was induced by retrograde filling cystometry at a rate of 20 mL/min [21] with an infusion pump containing sterile lactated Ringer's solution. EAS EMG during micturition was examined using Dantec® Keypoint® G4 EMG/NCS/EP Workstation (Figure 1). The area around the anus was wiped with alcohol. A 30 G silicone-coated stainless steel concentric needle electrode is 25 mm long (Natus™, EN9013S0012), serving as recording and reference electrodes were placed into the EAS, which was located 30° of the left lateral side at 1 cm distance parallel to the anal orifice. The separate reusable ground electrode is a circular disc with a diameter of approximately 30.5 millimeters, and it can be placed anywhere else on the electrical neutral tissue of the dog. The ground electrode reduces artifacts and prevents electrical interference and the transfer of any unwanted electrical charge [22]. In this study, the ground electrode was placed on the skin at the tail base, which is easy to apply for the position. We can secure the surface contact area of the ground electrode to the dog's body by enwrapping it with an adhesive bandage. The electrical activity of the EAS during retrograde filling cystometry was recorded using the KEYPOINT.NET 2.40 software program and focused on the micturition event. The feeding tube was removed from the dog after urine voiding was initiated.

### 2.5. Electrophysiological Bulbocavernosus Reflex

**Figure 2:**
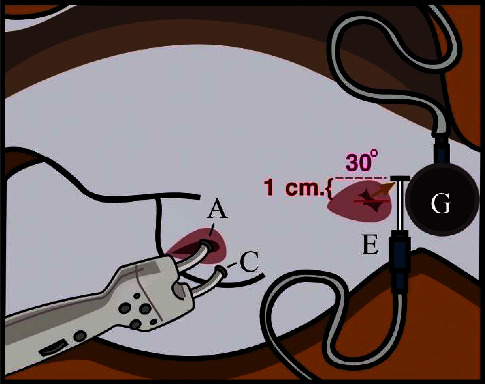
The position of the dog and electrode during the electrophysiological bulbocavernosus reflex (EBCR). A stimulator probe consists of two electrodes (an anode (A) and a cathode (C)). A concentric needle electrode was placed in the EAS (E), and the ground electrode was placed at the tail base (G).

The EBCR test was performed through a single-pulse electrical stimulation with a frequency of 1.5 Hz using the Dantec® Keypoint® G4 EMG/NCS/EP workstation, and data were analyzed using the KEYPOINT.NET 2.40 software program. The signal waveform was recorded when the dog was placed in left lateral recumbency. A surface stimulation electrode was placed on the clitoris to elicit the dorsal nerve of the clitoris, and a signal gel was applied to the clitoris for better conduction. A 30 G silicone-coated stainless steel concentric needle electrode (Natus™, EN9013S0012) and a separate reusable ground electrode were placed at the same positions with the EAS EMG examination (Figure 2). The electrical stimulus was slowly increased to meet the necessary stimulus threshold to obtain visible contraction of the EAS, which required a stimulus intensity of 3–35 milliampere. An average of 100 consecutive stimulations were performed and repeated to ensure reproducibility. The onset latency and mean baseline to peak amplitude were recorded.

### 2.6. Statistical Analysis

**Figure 3:**
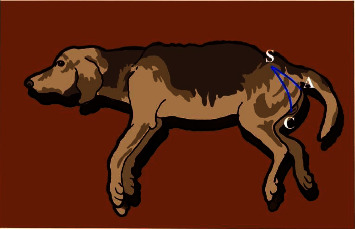
The blue line indicates the distance from the recording site to the stimulation site, which is referred to as the external anal sphincter-S1-clitoris. (A) Anal sphincter, (B) S1, and (C) clitoris.

Subject signalment, such as age, body weight, hind limb length, and EAS-S1-clitoris distance (Figure 3), was summarized as a mean and standard deviation. Data of EBCR onset latency values (ms) and EBCR mean amplitude values (*μ*V) were tabulated, tested for normality using an Anderson–Darling test, visualized using histograms, and tested for outliers using the Dixon–Reed and Tukey's tests. All normality tests, outlier tests, and reference interval computations were performed using reference value Advisor V 2.1 software [23]. Lower and upper limits of the reference interval of EBCR onset latency values and EBCR mean amplitude values were calculated using both standard and robust methods with untransformed and Box-Cox transformed data. Additionally, the reference intervals estimated using the nonparametric method were estimated depending on the number of samples. However, the sample size in the current study was very low (*n* = 10). The CLSI guideline suggests determining the reference intervals using a robust method, which requires only the symmetrical distribution of the data disregarding the Gaussian or normal distribution as required for the standard method [19]. EAS EMG during micturition was described as an electromyographic pattern.

## 3. Results

### 3.1. Study Population

Ten healthy spayed dogs with no breed restriction aged 2.36 ± 0.92 (range: 2–5) years with a mean body weight of 14.46 ± 2.31 (range: 11.3–18) kg were enrolled in the study. Their hind limb length was 53.57 ± 3.38 (range: 48–60) cm, and the EAS-S1-clitoris distance was 34.83 ± 0.83 (range: 34–36) cm. The data of dogs that participated in this study are shown in Table 1.

### 3.2. Electromyography of the External Anal Sphincter during Micturition

EAS EMG was performed in 10 healthy spayed female dogs. Complete cycles of urination were present in only seven dogs. The other three dogs showed only one waveform pattern at all times and when the pressure in the bladder was increased due to the increased amount of fluid infused beyond the bladder's capacity, urine overflows or drips out of the animal. Thus, a total of 34 complete cycles of EAS EMG during micturition were observed in seven subjects.

**Figure 4:**
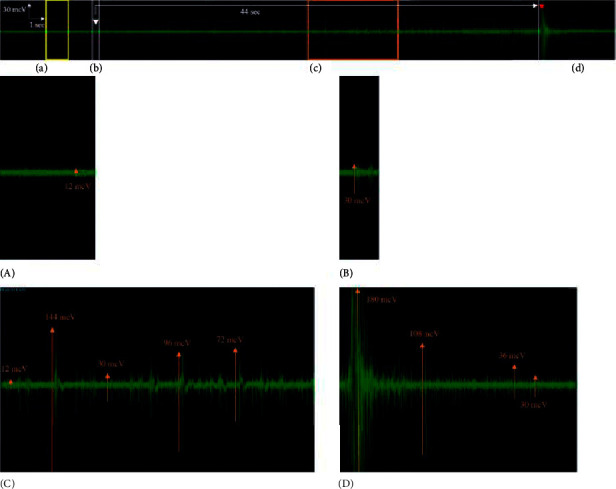
EAS EMG during micturition. (a) Before the voiding phase, low-amplitude high-frequency bursting is exhibited. (b) At the beginning of the voiding phase, medium-amplitude low-frequency bursting is exhibited. (c) During the voiding phase, oscillate medium- and/or high-amplitude low-frequency bursting with low-amplitude high-frequency bursting is exhibited. (d) At the end of the voiding phase, high-amplitude high-frequency bursting (guarding reflex) is exhibited. The white arrow indicates the initial urine leakage and the red arrow indicates the end of urination. EAS, external anal sphincter; EMG, electromyography. (A) Zoomed window of the yellow square from (a) phase; the most prominent pattern is a low-amplitude high-frequency bursting. (B) Zoomed window from the (b) phase, a medium-amplitude low-frequency bursting at the beginning of voiding. (C) Zoomed window of the orange square from (c) phase, oscillating medium- and high-amplitude low-frequency bursting between low-amplitude high-frequency bursting during the pulsatile flow of urine. (D) Zoomed window from (d) phase; the most prominent pattern is a high-amplitude high-frequency bursting.

Micturition could be divided into the following four phases: before voiding, beginning of voiding, during voiding, and after voiding. Twenty-five out of 34 data EAS EMG showed a similar pattern, including low-amplitude high-frequency bursting before voiding, medium- or high-amplitude low-frequency bursting at the beginning of voiding, oscillate medium- and/or high-amplitude low-frequency bursting with low-amplitude high-frequency bursting during voiding, and reappearance of high-amplitude high-frequency bursting that is identified as a guarding reflex at the end of voiding (Figure 4).

### 3.3. Electrophysiological Bulbocavernosus Reflex

**Figure 5:**
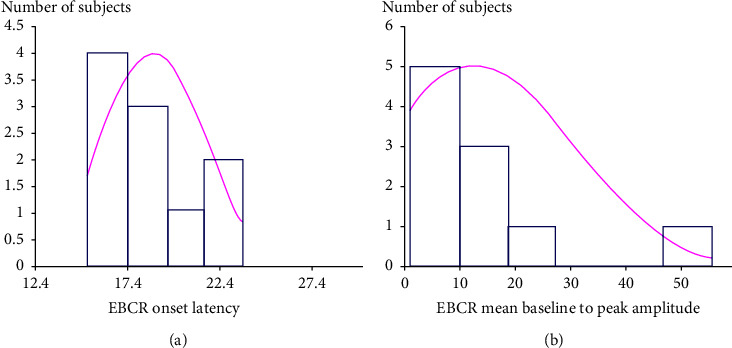
Histograms show the two tested analyte distributions. EBCR onset latency (a) exhibits a Gaussian distribution, whereas the EBCR mean baseline to peak amplitude (b) exhibits a non-Gaussian distribution.

The EBCR test was successfully performed in 10 spayed female canine subjects and was repeated to ensure reproducibility. The test of normality according to Anderson–Darling [16] demonstrated that EBCR onset latency revealed a Gaussian distribution (*P* > 0.05), while the EBCR mean baseline to peak amplitude revealed a non-Gaussian distribution (*P* < 0.05) (Figure 5).

The reference intervals for EBCR onset latency determined parametrically with the standard and robust methods were 12.34–25.1 and 12.07–25.07 ms, respectively. The reference intervals for EBCR onset latency determined parametrically with standard and robust methods after Box-Cox transformation were 13.92–27.3 and 13.85–27.44 ms, respectively. The overall reference intervals of EBCR onset latency agreed with the International Federation of Clinical Chemistry (IFCC) and the Clinical Laboratory Standard Institute (CLSI) recommendations (Table 2).

**Figure 6:**
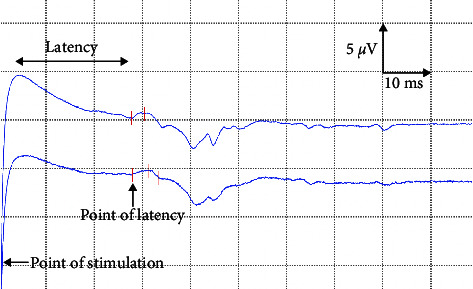
An example of electrophysiological bulbocavernosus reflex (EBCR) in a healthy spayed female dog. Two traces were obtained from the same subject to ensure the reproducibility of the response.

All EBCR compound muscle action potentials displayed a similar form, starting with a negative peak, which departed from baseline to upward (Figure 6). The reference intervals for the EBCR mean baseline to peak amplitude determined parametrically with the standard and robust methods were −25.82 to 51.98 and −32.16 to 47.39 *μ*V, respectively. The reference interval obtained with the standard method did not follow the IFCC-CLSI recommendation as the data were not normally distributed. On the other hand, the distribution of data was symmetric, and the reference interval estimated by the robust method could be estimated. However, the reference interval from the robust method should be used with caution or not used because possible outliers were detected. Moreover, the data could not be transformed with Box-Coxtransformation (Table 3).

## 4. Discussion

To the best of our knowledge, this is the first study reporting the electromyographic pattern of EAS EMG during micturition in a canine model. The characteristics of EAS EMG in spayed female dogs were different from those of the previous study in humans and rats [5, 8]. We observed baseline low-amplitude high-frequency bursting before the voiding phase, showing a low basal EAS activity. The electrical activity of EAS increased concomitantly with a sudden increase in intravesical pressure at the beginning of the voiding phase as it contracts to prevent stool leakage (guarding reflex) while EUS was relaxed to evacuate urine from the bladder. During the voiding phase, oscillate medium- and/or high-amplitude low-frequency bursting with a low-amplitude high-frequency bursting was displayed, which synchronized with the pulsatile flow of urine. The reappearance of the high-amplitude high-frequency bursting of EAS EMG indicated a contraction and reappearance of a strong guarding reflex at the end of urination.

There are several interesting points. First, the number of complete data cycles of EAS EMG during micturition varied because each dog has a different temperament and tolerance. Performing EAS EMG during micturition and/or the ECBR test in a female dog was difficult because the dog could not stay still during the procedure. Hence, performing light anesthesia with the dog was needed. Dexmedetomidine and propofol were used in this study; however, interferences were found from several factors. The anesthesia plane of canine subjects always becomes lighter when micturition occurs. Koyama et al. [24] support this observation by revealing the potential of urinary bladder distension to induce the transition of an anesthesia state from deep sleep to light sleep, which is mediated by noradrenergic and cholinergic neurons in the brainstem. Therefore, three dogs, with failed recordings of the complete cycle of micturition, woke up when the micturition occurred. We were unable to restrain them, causing safety concerns to the veterinarians and damage to the equipment, leading to propofol administration to anesthetize the dogs. Consequently, it resulted in one waveform pattern: a small amplitude with high frequency throughout the micturition cycle and urine overflow. Altogether, 34 data had complete micturition cycles; of these, only 25 data showed a similar pattern, whereas the other nine data showed different patterns, which were caused by the interference of the internal and external factors, including shivering of body, spasticity of hind limbs, tucked tail, and movement of silicone-coated stainless steel concentric needle electrode. Moreover, because of ethical reasons, we performed only six complete micturition cycles per dog. If we found an unusual electrical activity of the EAS after a repetitive micturition cycle examination, the experiment would be immediately stopped.

It is important to recognize a normal finding on sphincter EMG as it has the potential to rule out neurological causes of the patient's voiding dysfunction because an abnormal finding of sphincter EMG may signify that future diagnosis is needed [1, 25]. The function of the sphincter during bladder filling and voiding can be demonstrated by EMG [26]. In human medicine, the evaluation of sphincter EMG during micturition is essential in patients with voiding disorder and bladder outlet obstruction as both can be caused by anatomical outflow obstruction or functional abnormalities [1, 27].

The current study also provided a reference interval of EBCR onset latency in healthy spayed dogs. Based on the IFCC-CLSI recommendation, when the sample size is <120, a robust method after data transformation that is close to Gaussian or normal is preferable to establish the reference interval [23]. The reference intervals of EBCR onset latency of 13.85–27.44 ms in our study were similar to both male and female Beagles' EBCR latency reported in previous studies [12, 13]. This study demonstrated that mongrel dogs have different genetics, but age and body weight nearly showed resembling EBCR onset latency. All EBCR compound muscle action potentials started with a negative sharp wave, which tapers from the baseline in the upward direction, showing an upturned bell-shaped curve and confirming that the electrode is on the muscle innervated by the stimulated nerve. The shape of the EBCR compound muscle action potential in our study was similar to that of the previous study [11]. However, we were unable to transform the EBCR mean amplitude data obtained from the subjects using the Box-Cox transformation. In fact, the amplitude could not be deducted, but when we calculated the normal range using the program (approximate mean ± 2SD), some negative values were observed in the normal range. The mean amplitude of EBCR by both the standard and robust methods with untransformed data also had a high standard deviation, which was not in agreement with the IFCC-CLSI recommendation. Granata et al. [11] assessed the EBCR in 105 men and demonstrated that the EBCR mean baseline to peak amplitude had a high standard deviation. They suggested that the EBCR mean baseline to peak amplitude may not be a reliable parameter for assessing the normal function of EBCR. Future studies focused on this topic are needed to confirm this assumption. The EBCR test has the potential to early detect disorders in the pudendal nerve and lower sacral segment [28, 29]. Moreover, it helps distinguish upper motor neuron lesions from lower motor neuron lesions, which are beneficial for therapeutic implications and prognosis [30].

In human medicine, both sphincter EMG during micturition and the EBCR tests are performed in awake and unanesthetized or unsedated patients. Anesthetic agents are known to have a confounding effect on the urological function, as they can generally increase bladder capacity, decrease maximal intravesical pressure, and depress the micturition response [31–35]. However, anesthesia is usually required in animal models due to the non-cooperation of conscious veterinary patients. In feline models, dexmedetomidine was proved to have the most similar urological outcomes as those in awake or decerebrate animals, whereas propofol had the most obvious change from the awake stage, which is related to the loss of function and initiation of the voiding reflex [36]. In contrast, a previous study on a canine model demonstrated that propofol provided a safe and stable light plane of anesthesia and also resulted in good reproducibility of urological data [21]. The present study utilized anesthesia with a combination of propofol and dexmedetomidine, as we found that dexmedetomidine alone was unable to maintain the suitable plane of anesthesia while experimenting when we encountered aggressive dogs. It is important to maintain a suitable plane of anesthesia as the micturition reflex and electrical activity of EAS are easily influenced by the depth of anesthesia. Li et al. [37] generally support this opinion. In their experiment using a feline model to test the effect of the level of consciousness on the urodynamic procedure, sphincter EMG during micturition could be strongly affected by anesthetic depth, as a lighter anesthesia plane was reported to be associated with a more awake-like bladder response.

EBCR onset latency in our study that used propofol combined with dexmedetomidine was similar to that in a previous study that used ketamine combined with xylazine as standard anesthesia protocol in canine EBCR [12, 13]. Empirically, propofol caused less suppression of the cortical somatosensory evoked potentials, and dexmedetomidine did not influence the nerve conduction velocity [38, 39].

Adult neurogenic lower urinary tract dysfunction could be caused by damage at any level of the micturition pathway, but sphincter EMG during micturition and especially the EBCR test are very limited to the sacral segment. EMG activity can also be influenced by external and internal factors, such as shivering of the body, spasticity of hind limbs, tucked tail, and movement of the needle; thus, interpretations should be conducted with caution and video recording of the subjects during the experiment is necessary for sphincter EMG interpretation [26]. To perfectly localize the lesion of the neurogenic lower urinary tract dysfunction, further diagnosis, such as urodynamic tests, imaging examinations, and so on, can provide useful information for diagnosis [27]. However, sphincter EMG during micturition can be used as a screening test for neurogenic lower urinary tract dysfunction. According to the existing literature, sphincter EMG combined with the EBCR test is one of the neurophysiological tests with the greatest clinical utility in patients with suspected adult neurogenic lower urinary tract dysfunction at the sacral segment [11]. The application of sphincter EMG and EBCR tests as novel investigative tools for the neurogenic assessment of the lower urinary tract dysfunction of dogs may aid clinical practice and diagnosis.

A major limitation of this study was the small number of dogs enrolled. We tried to compensate for this by recording multiple micturition cycles per dog and by using a robust method after data transformation when calculating the EBCR test results. Additionally, we only used one anesthetic protocol, which utilized a combination of propofol and dexmedetomidine. To the best of our knowledge, no study has compared the effect of anesthetic drugs on neuro-urology in a canine model. Therefore, the use of different anesthetic drugs may show different results.

## 5. Conclusions

This study described the method and provided the electrographic pattern of EAS EMG during micturition and the reference intervals of EBCR onset latency in spayed female dogs, which serve as baseline information to help veterinarians differentiate healthy from diseased dogs. Further studies should compare normal dogs and dogs with lower urinary tract abnormalities at different lesion locations.

## Figures and Tables

**Table 1:** Data from 10 spayed female dogs.

Dog no	Age (years)	Body weight (kg)	Body condition score	Left hindlimb length (cm)	The EAS-S1-clitoris distance (cm)
1	3	14	6/9	53	35
2	2	11.3	5/9	49	35
3	2	18	5/9	60	36
4	2	18	5/9	55	34
5	2	17	5/9	56	36
6	2	14	5/9	54	34
7	2	14	4/9	57	34
8	2	12	5/9	50	34
9	5	15	6/9	50	36
10	2	13	5/9	52	35

**Table 2:** Computations made with a series of 10 EBCR onset latency values (ms).

Method	Untransformed data		Box-Cox transformed data		Nonparametric
	Standard	Robust	Standard	Robust	
*N*	10	10	10	10	10
Mean	18.72		1.47		
Median	18.40	18.57	1.47	1.47	
SD	2.69	2.87	0.06	0.06	
Minimum	15.2	15.2	1.39	1.39	
Maximum	23.5	23.5	1.56	1.56	

*λ*_1_ coefficient Box-Cox			−5.185	−5.185	
*λ*_2_ coefficient Box-Cox			−0.484	−0.484	
*P* value Anderson–Darling	0.685		0.923		
Symmetry test for robust		0.746		0.998	

Outliers Dixon					
Outliers Tukey	0	0	0	0	
Suspect data Tukey	0	0	0	0	

Lower limit of reference interval	12.34^a^	12.07^a^	13.92^a^	13.85^a^	
Upper limit of the reference interval	25.10^a^	25.07^a^	27.30^a^	27.44^a^	
90% CI for lower limit	9.98		12.65		
	15.10		15.59		
90% CI for upper limit	22.22		22.46		
	27.81		34.50		

*Comments*					
The sample size is too small (*n* < 40) to compute a nonparametric reference interval. Data should be analyzed with the nonparametric method. As an alternative, the robust method with a Box-Cox transformation may be used after checking the symmetry of the distribution. The robust method with a Box-Cox transformation of the data gives the following reference interval: [13.85; 27.44]. The sample size is too small (*n* < 20) to compute the CIs for the limits of the reference interval obtained with the robust method with Box-Cox transformation.					

^a^In agreement with the IFCC-CLSI recommendation.

**Table 3:** Computations made with a series of 10 EBCR mean amplitude values (*μ*V).

Method	Untransformed data		Box-Cox transformed data		Nonparametric
	Standard	Robust	Standard	Robust	
*N*	10	10	10	10	10
Mean	13.079				
Median	7.750	7.612			
SD	16.395	17.582			
Minimum	1.11	1.11			
Maximum	54	54			

*λ*_1_ coefficient Box-Cox					
*λ*_2_ coefficient Box-Cox					
*P*-value Anderson–Darling	0.009				
Symmetry test for robust		0.254			

Outliers Dixon	Max	Max			
Outliers Tukey	0	0			
Suspect data Tukey	1	1			

Lower limit of the reference interval	−25.82^c^	−32.16^b^			
Upper limit of the reference interval	51.98^c^	47.39^b^			
90% CI for lower limit					
90% CI for upper limit					

*Comments*					
The sample size is too small (*n *<* *40) to compute a nonparametric reference interval. Data should be analyzed with the nonparametric method. As an alternative, the robust method with a Box-Cox transformation may be used after checking the symmetry of the distribution. The robust method with a Box-Cox transformation of the data gives the following reference interval: [0; 0]. The sample size is too small (*n *<* *20) to compute the CIs for the limits of the reference interval obtained with the robust method with Box-Cox transformation.					

^b^Use with caution or avoid using because possible outliers are detected. ^c^The distribution is not Gaussian.

## Data Availability

The data used in this study are available upon request from the corresponding author.
